# *In vivo* therapeutic exploring for *Mori folium* extract against type 2 diabetes mellitus in rats

**DOI:** 10.1042/BSR20210977

**Published:** 2021-12-08

**Authors:** Kaibo Lyu, Wei Yue, Junhua Ran, Yunjuan Liu, Xueliang Zhu

**Affiliations:** 1 College of Environmental and Biological Engineering, Wuhan Technology and Business University, Wuhan 430065, Hubei, P.R. China; 2 Department of Nutrition, The First Affiliated Hospital of Xinxiang Medical University, Weihui 453100, Henan, P.R. China; 3 I Faithfood Technology (Beijing) Co., Ltd, Beijing 100007, P.R. China; 4 State Key Laboratory of Advanced Technology for Materials Synthesis and Processing, Wuhan University of Technology, Wuhan, Hubei 430070, P.R. China

**Keywords:** glucose metabolism, insulin resistance, Mori folium aqueous extract, oxidative stress, type 2 diabetes mellitus

## Abstract

**Background:** The study was aimed to investigate the potential therapeutic effect of *Mori folium* aqueous extracts (MFAE) on type 2 diabetes mellitus (T2DM) *in vivo*.

**Methods and results:** A rat model of T2DM was established with the combination of high sugar and high-fat diet (HSFD) and streptozotocin (STZ). The T2DM rats were administrated with low (2 g.kg^−1^) and high (5 g.kg^−1^) doses of MFAE for 60 consecutive days. The biochemical indices of glucose metabolism disorders, insulin resistance and oxidative stress were observed. The results indicated that MFAE significantly promoted the synthesis of hepatic glycogen, reduced the levels of fasting blood glucose and fasting blood insulin, and improved the insulin sensitivity index (ISI). MFAE administration also remarkably increased the levels of superoxide dismutase (SOD) and reduced the levels of malondialdehyde (MDA).

**Conclusion:** MFAE showed a therapeutic effect on T2DM with the bioative effect of improve glucose metabolism disorders, decrease insulin resistance, and ameliorate the antioxidative ability.

## Introduction

Type 2 diabetes mellitus (T2DM) is a complicated disease. With the rapid increase in the number of T2DM patients in the worldwide, it has become one of the most serious challenges for global public health [1–3]. Modern medicine believes that diabetes is an endocrine and metabolic disease characterized by glucose metabolism disorder caused by relative or absolute insufficiency of insulin secretion in the body [1,2]. It is characterized by hyperglycemia and glycosuria, as well as disorders of fat and protein, which often lead to serious complications, such as eye and neuropathy, coronary heart disease, cerebrovascular disease etc. [3,4]. Although metformin [5] and sulfonylureas [6,7] are effective in the treatment of T2DM in clinic, they have some problems such as low efficacy, low durability and toxic side effects, which cannot fundamentally eliminate the causes and prevent the occurrence of complications [8]. Therefore, the research and development of drugs which can regulate blood glucose and prevent complications with low toxicity and side effects has become an important direction and hotspot of T2DM drug development.

*Mori folium* are dried leaves of *Morus alba* L. (*Moraceae*, Morus L.). It is one of the commonly used traditional Chinese medicines in clinic [9,10]. Modern studies have shown that the alkaloid constituents in *Mori folium*, represented by 1-deoxynojirimycin (1-DNJ), can significantly regulate blood glucose; flavonoids and polysaccharides in *Mori folium* can regulate blood glucose and prevent complications [11]. In addition, *Mori folium* still contains a variety of functional components, such as phytosterols, trace elements, vitamins, amino acids [12,13]. Pharmacological evaluation showed that *Mori folium* exerts variety of pharmacological effects, such as hypoglycemia, hypotension, antibacterial, antiviral activities [14–16], anti-tumor, anti-inflammatory, anti-oxidant, and anti-obesity etc. [11,14,17,18].

To the best of our knowledge, there is no study on the therapeutic effect and potential bioactivity constituents of *Mori folium* aqueous extracts (MFAE) on T2DM. In this work, as a part of our research on novel biologically active substances for the prevention and treatment of T2DM from traditional medical resources, we investigated the potential therapy effect and potential bioactivity constituents of MFAE on high sugar and high-fat diet (HSFD) combined streptozotocin (STZ) induced T2DM.

## Materials and methods

### Materials and reagents

Metformin was purchased from Selleck Chemicals (U.S.A.). Superoxide dismutase (SOD), malondialdehyde (MDA), glucose determination kit, liver glycogen detection kit were obtained from Nanjing Jiancheng Bioengineering Co., Ltd. (China). Rat insulin ELISA kit was purchased from Mercodia Co., Ltd. (Sweden). STZ was purchased from Sigma (U.S.A.). The formula of HSFD containing 59% common feed, 10% lard, 10% yolk powder, 20% sucrose, and 1% cholesterol was purchased from Beijing Keao Xieli Feed Co., Ltd. (China).

### Plant material and preparation of MFAE

The dried leaves of *Morus alba* were obtained from Nanjing Zelang Pharmaceutical Technology Co., Ltd. (Nanjing, China) and were authenticated by Yunjuan Liu, I Faithfood Technology (Beijing) Co., Ltd.

Firstly, *Mori folium* was cut into small pieces, and ground to a fine powder. Then, 5 kg *Mori folium* powder was taken with 40 l distilled water, and boiled for 3 h. The water extracts were filtered twice with three Whatman filter paper (Sigma, U.S.A.) to remove the insoluble materials, the filtrate was lyophilized and stored at −80°C before use. The administration of MFAE was dissolved with saline (200 and 500 mg/ml, respectively) prior to use.

### High-performance liquid chromatography analysis of MFAE

The main components of MFAE were analyzed and semi-quantitated by high-performance liquid chromatography analysis (HPLC) method with the reference standard of compounds 1–4, which were isolated previously from the extracts of MFAE by author; structures were elucidated by comparison of spectral data (^1^H NMR and ^13^C NMR) with the literature data. The purity of compounds 1–4 was above 98% by LC analysis based on a peak area normalization method. The semi-quantitated HPLC analysis method was done according to reference [19,20].

The HPLC System Waters 2695 Alliance combined with Waters 2998 PDA detector was used for qualitative analysis of MFAE. The SHIMADZU C18 LC column (150 × 4.6 mm, 5 μm), with a column temperature of 30°C, was applied. The gradient elution process with the mobile phase (A, 1% formic acid in water; B, acetonitrile) is as follows: 0–5 min with 15–21% of mobile phase B, 5–22 min with 21–50% of mobile phase B, 22–32 min with 50–80% of mobile phase B, 32–40 min with 80–98% of mobile phase B, 40–45 min with 98% of mobile phase B, 45–47 min with 98 to 15% of mobile phase B, 47–55 min with 15% of mobile phase B. The flow rate was 1 ml/min.

### Animals

Male Sprague–Dawley (SD) rats with the body mass of 200 ± 10 g of 6 weeks of age were supplied by the Center of Experimental Animal at Wuhan University of Technology (Wuhan, China). They were housed in specific pathogen-free (SPF) grade animal rooms at a controlled temperature (20 ± 2°C) and controlled humidity (45 ± 5%) under 12-h of light/dark cycles (lights on from 7:00 a.m. to 7:00 p.m.). All the rats were provided with food and water *ad libitum* and quarantined for at least 3 days before experiment. The diabetic rats were fed with HSFD (containing 59% common feed, 10% lard, 10% yolk powder, 20% sucrose, and 1% cholesterol was purchased from Beijing Keao Xieli Feed Co., Ltd., China) for 4 weeks. The rats received humane care, the experimental procedures were performed according to National Institutes Health Guidelines of U.S.A. (National Research Council of U.S.A., 1996). The experiment was carried out at Wuhan University of Technology and was approved by the University ethical regulations with the protocol number ZXL-0013.

### Experimental design

Fifty male SD rats were randomly divided into untreated group and diabetic group. The diabetic rats were fed with HSFD for 4 weeks, then injected with 30 mg.kg^−1^ STZ through tail vein. One week later, only the rats with fasting blood glucose concentration higher than 16.7 mmol.l^−1^ were selected as the diabetic rats. The untreated control group rats were fed with normal diet (100% common feed). Then the selected diabetic rats were randomly divided into four groups (*n*=10): diabetic group, metformin positive control group (300 mg.kg^−1^), the MFAE low-dose group (2 g.kg^−1^), and MFAE high-dose group (5 g.kg^−1^). Oral administration was performed once a day for 60 consecutive days. The untreated and diabetic groups received equivalent amounts of saline once a day for the same duration of time. At the end of the experiment, rats were fasted for 12 h, the mice were killed by cervical dislocation and the blood was collected from hepatic portal vein of each animal, centrifuged at 4°C, 1800×***g***, 15 min; the serum was then taken for the determination of biochemical parameters, the liver was taken for hepatic glycogen analysis. The pancreas was taken and fixed with 10% formaldehyde solution for histopathological examination.

### Detection of hepatic glycogen levels in liver tissue

Hepatic glycogen was detected by anthron method according to the report of Seifter et al. [21]. Briefly, liver tissue snip (25 mg) was boiled with 30% KOH (1 ml) for 20 min. The tissue digested was cooled, then 95% ethanol (1.25 ml) was added and centrifuged at 3000 rpm for 15 min. The supernatant was discarded and the tube was allowed to be drained on tissue paper. The sedimented glycogen pellets of the tube were dissolved in 2 ml water, then treated with anthrone reagent (1:2 v/v), boiled for 10 min to develop color. The optical density was measured at 620 nm within 2 h. The glycogen content was analyzed relatively from the calibration curve.

### Biochemical assays

The biochemical parameters of fasting blood sugar and fasting serum insulin were measured according to the protocol of the commercial kit [22]. The insulin sensitivity index (ISI) [23] and insulin resistance index (IRI) [24] were calculated:

$$\text{ISI }=\;\text{ln}\left[\frac{1}{\left(\text{fasting blood sugar }\times \;\text{fasting blood insulin}\right)}\right]$$

$$\text{IRI}=\frac{\left(\text{fasting blood sugar }\times \;\text{fasting blood insulin}\right)}{22.5}$$

SOD and MDA levels were measured by commercial kit from Jiancheng Biological Engineering Institute (China) following manufacturer’s instructions.

### Histopathological analysis

The tissues of pancreas fixed in 10% formalin buffer were dehydrated, respectively, and then embedded in paraffin and cut into sections of 5-µm thickness according to the routine procedure. Then the tissue sections were stained with Hematoxylin and Eosin (H&E) for routine histopathological examination. The image of the tissue histopathology sections was captured under a light microscope (Olympus BX-50 Microscope, Leica Microsystems, Germany) at 200× magnification.

### Statistical analysis

Statistical data are presented as means ± SD. All statistical comparisons were performed with Dunett’s *t* test of one-way ANOVA by SPSS version 17.0 software.

## Results

### Chemical constituents analysis

**Figure 1:**
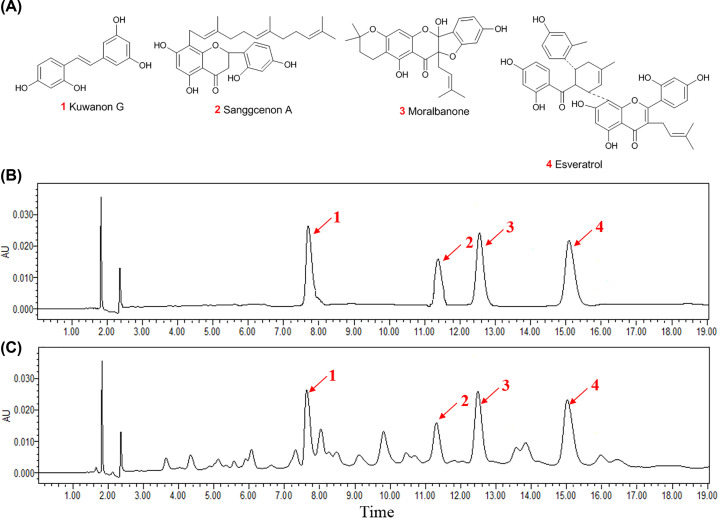
Chemical components analysis of MFAE (**A**) Chemical structures of compounds 1–4. (**B**) HPLC chromatograms of compounds 1–4. (**C**) HPLC chromatograms of MFAE.

The potential bioactive components of MFAE were analyzed and semi-quantitated by HPLC (Figure 1). In HPLC analysis of MFAE, we identified four high content compounds (**1**–**4**) by comparing with the reference standard compounds, which were isolated and elucidated from MFAE by author. In addition, the four main compounds were semi-quantitated and the contents of these compounds in MFAE are shown in Table 1. The previous study showed that these flavones were the main components of MFAE, and showed many pharmacological effects, such as anti-oxidant, anti-tumor, anti-inflammation, regulating the immune system, and hypoglycemic effect [19,20].

**Table 1:** Constituents content of the four identified compounds in MFAE (mg/g)

Sample	Compound 1	Compound 2	Compound 3	Compound 4
MFAE	6.03	7.45	15.33	18.92

### Effect of MFAE on body changes during the experiment

**Figure 2:**
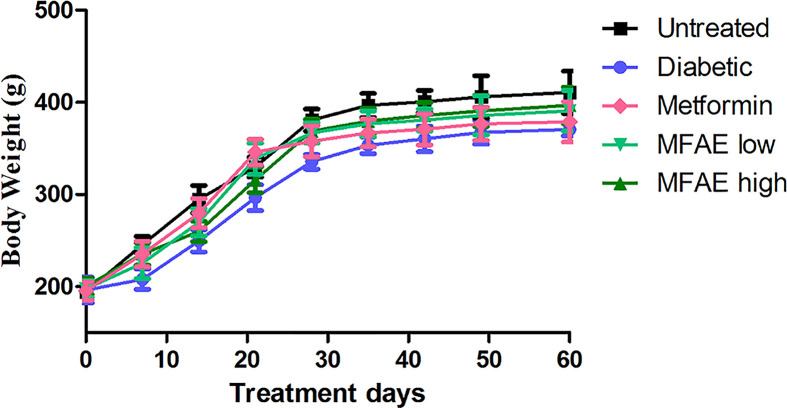
Effect of MFAE on body weight in type 2 diabetic rats (*n*=10) Untreated group (saline), Diabetic group (saline), Metformin group (300 mg.kg^−1^), MFAE low-dose group (2 g.kg^−1^), MFAE high-dose group (5 g.kg^−1^). The data are given as mean ± SD.

In the whole animal experiment, all the rats survived, and there was no observed toxicity to rats in each group. The body weight in each group during the whole experiment is shown in Figure 2, and there was no significant differences between each group.

### Effect of MFAE on levels of the fasting blood glucose in T2DM rats

**Figure 3:**
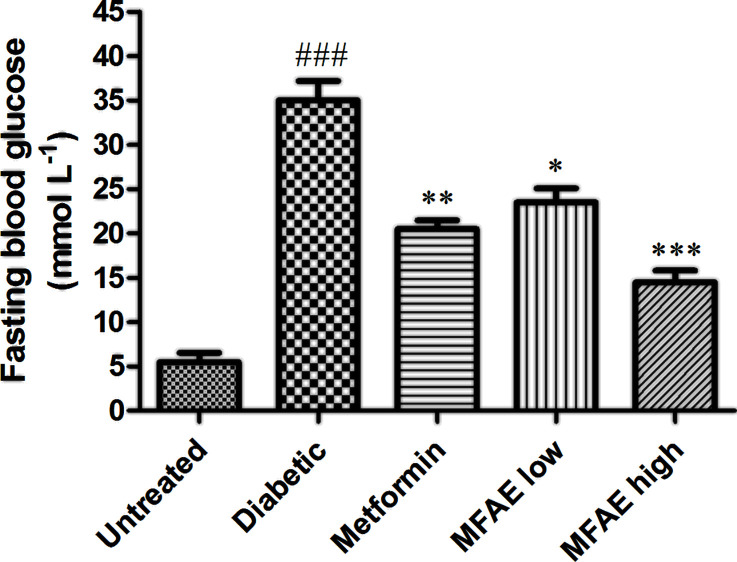
Effect of MFAE on fasting blood glucose in type 2 diabetic rats (*n*=10) Untreated group (saline), Diabetic group (saline), Metformin group (300 mg.kg^−1^), MFAE low-dose group (2 g.kg^−1^), MFAE high-dose group (5 g.kg^−1^). The data are given as mean ± SD. Significant difference: ^###^*P*<0.001 vs. untreated group, *P<0.05, **P<0.01, ***P<0.001 vs. diabetic group.

Effect of MFAE on the levels of fasting blood sugar in T2DM rats is shown in Figure 3. Compared with the untreated group, the fasting blood glucose in the diabetic group increased significantly (*P*<0.001). In MFAE low (2 g.kg^−1^) and high dose (5 g.kg^−1^) treated groups, the level of the fasting blood glucose was dramatically reduced, especially in the high-dose group (*P*<0.05), which was almost reduced to 65% compared with the diabetic group. The metformin treated group decreased approximately 38% of fasting blood glucose levels as the previous study reported (*P*<0.01).

### Effect of MFAE on levels of the hepatic glycogen in T2DM rats

**Figure 4:**
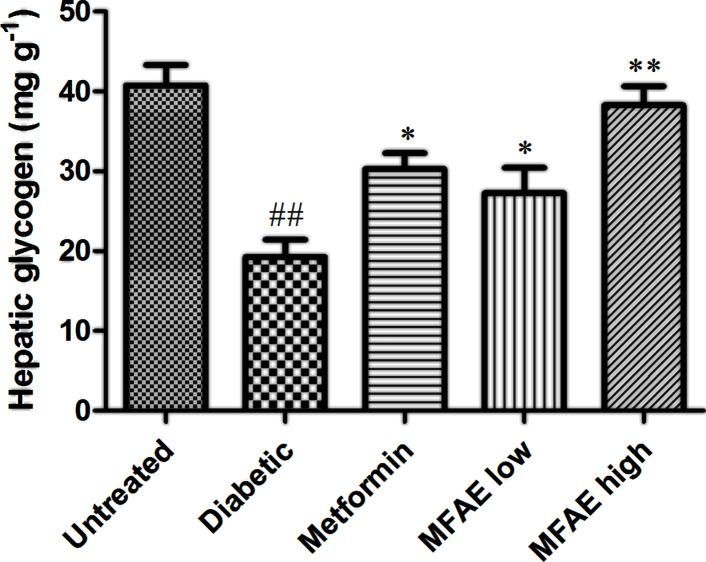
Effect of MFAE on hepatic glycogen in type 2 diabetic rats (*n*=10) Untreated group (saline), Diabetic group (saline), Metformin group (300 mg.kg^−1^), MFAE low-dose group (2 g.kg^−1^), MFAE high-dose group (5 g.kg^−1^). The data are presented as mean ± SD, ^##^*P*<0.01 vs. untreated group, *P<0.05, **P<0.01 vs. diabetic group.

The hepatic glycogen content in liver was detected by anthron method. In diabetic group, the level of hepatic glycogen was decreased for approximately 61%, compared with untreated group (*P*<0.01, Figure 4). In MFAE high-dose (5 g kg^−1^) group, it was increased at approximately 48.8% of hepatic glycogen compared with diabetic group (*P*<0.01, Figure 4). While MFAE low-dose group (2 g.kg^−1^) also significantly increased the levels of hepatic glycogen (*P*<0.05, Figure 4), compared with the diabetic group.

### Effect of MFAE on levels of the fasting blood insulin in T2DM rats

**Figure 5:**
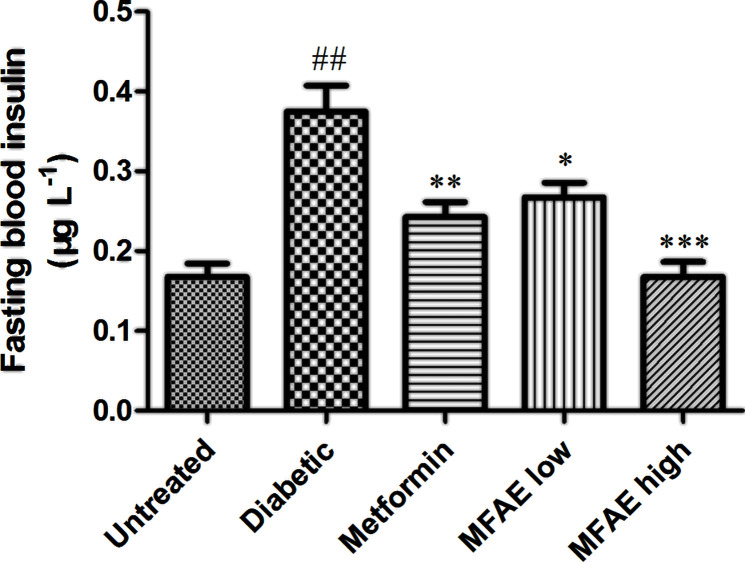
Effect of MFAE on fasting blood insulin in type 2 diabetic rats (*n*=10) Untreated group (saline), Diabetic group (saline), Metformin group (300 mg.kg^−1^), MFAE low-dose group (2 g.kg^−1^), MFAE high-dose group (5 g.kg^−1^). The data are presented as mean ± SD, ^##^*P*<0.01 vs untreated group, *P<0.05, ***P<0.001 vs diabetic group, ns: not significant.

The effect of MFAE on fasting blood insulin levels in T2DM rats is shown in Figure 5. Compared with the untreated group, the fasting blood insulin content in diabetic group significantly increased (*P*<0.01, Figure 5). The fasting blood insulin in MFAE-treated group decreased significantly by 30% in the low-dose (2 g.kg^−1^) MFAE group (*P*<0.05, Figure 5), and by 65% in the high-dose (5 g.kg^−1^) MFAE group (*P*<0.001, Figure 5). Metformin-treated group also had a significant decrease in fasting blood insulin (*P*<0.01, Figure 5), compared with the diabetic group.

### Effect of MFAE on levels of the ISI and IRI in T2DM rats

**Figure 6:**
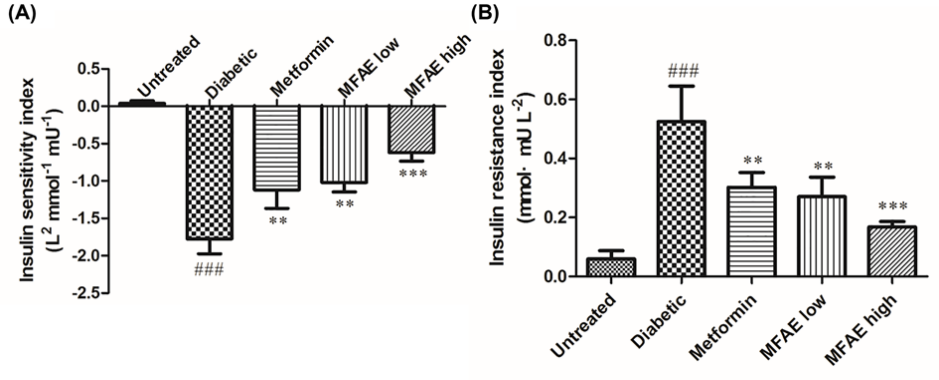
Effect of MFAE on insulin sensitivity and resistance index in type 2 diabetic rats (*n*=10) (**A**) ISI, (**B**) IRI. Untreated group (saline), Diabetic group (saline), Metformin group (300 mg.kg^−1^), MFAE low-dose group (2 g.kg^−1^), MFAE high-dose group (5 g.kg^−1^). The data are presented as mean ± SD, ^###^*P*<0.001 vs Untreated group, **P<0.01, ***P<0.001 vs diabetic group.

The effect of MFAE on insulin sensitivity in T2DM rats is shown in Figure 6A, while the IRI is shown in Figure 6B. Compared with the untreated group, the ISI of diabetic group was significantly decreased, while the IRI of diabetic group was significantly increased (*P*<0.001, Figure 6). Compared with the diabetic group, the ISI of MFAE group increased by 39% in MFAE low-dose group (2 g.kg^−1^) and by 67% in MFAE high-dose group (5 g.kg^−1^) (*P*<0.01, *P*<0.001, Figure 6). In addition, the IRI of MFAE group was significantly decreased compared with the diabetic group (*P*<0.01, Figure 6B).

### Effect of MFAE on SOD and MDA levels in T2DM rats

Table 2 shows the effect of MFAE on SOD activity and MDA content. In the diabetic group, the serum levels of SOD were significantly lower than the untreated group (*P*<0.01), whereas in MFAE-treated groups the levels of SOD were significantly increased compared with the diabetic group, especially in the high-dose MFAE (5 g.kg^−1^) group (*P*<0.001). Metformin-treated group also significantly increased the levels of SOD (*P*<0.01). In addition, serum MDA content markedly increased in the diabetic group, compared with the untreated group (*P*<0.01). The serum content of MDA in MFAE-treated group significantly decreased compared with the diabetic group (*P*<0.05). Compared with the diabetic group, metformin-treated group significantly decreased the content of MDA in serum (*P*<0.01).

**Table 2:** Effect of MFAE on SOD and MDA of diabetic rats

Group	Dosage	SOD (U.ml^−1^)	MDA (mmol.l^−1^)
Untreated	-	318.5 ± 20.1	9.6 ± 1.2
Diabetic	-	97.1 ± 10.3^1^	24.2 ± 2.1^1^
Metformin	300 mg.kg^−1^	200.3 ± 8.6^3^	13.5 ± 1.6^3^
MFAE low-dose	2 g.kg^−1^	175.6 ± 13.9^3^	15.3 ± 1.7^2^
MFAE high-dose	5 g.kg^−1^	284.4 ± 18.3^4^	10.4 ± 2.4^4^

Data are expressed as mean ± SD for each group (*n*=10).

Significant differences: ^1^*P*<0.01 vs. untreated group. ^2^*P*<0.05, ^3^*P*<0.01, ^4^*P*<0.001 vs*.* diabetic group.

### Histopathological changes in pancreatic tissue of T2DM rats

**Figure 7:**
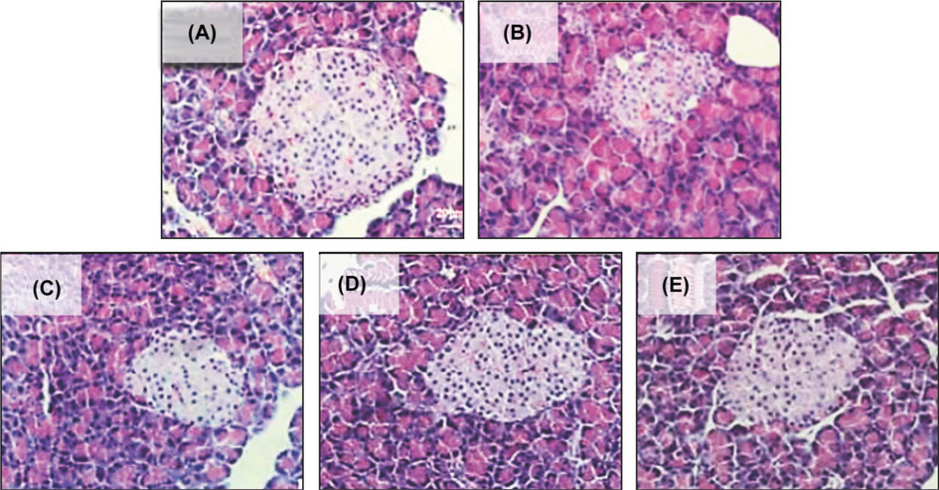
Effect of MFAE on pancreas in type 2 diabetic rats by H&E staining (*n*=10, 200×) (**A**) Untreated group (saline), (**B**) Diabetic group (saline), (**C**) Metformin group (300 mg.kg^−1^), (**D**) MFAE low-dose group (2 g.kg^−1^), (**E**) MFAE high-dose group (5 g.kg^−1^).

The histopathological analysis of pancreas in T2DM rats is shown in Figure 7. The morphology of islets in the untreated control group was complete, the boundary between islets and exocrine glands was clear, and the number of cells in islets was large and uniform (Figure 7A). In diabetic group, the morphology of islets was irregular, the boundary between islets and exocrine glands was blurred, the cytoplasm of islets was reduced, and some of the cytoplasm of islets had vacuolar degeneration, compared with untreated group (Figure 7B). Metformin treatment significantly improved the pancreas pathological character, the boundary between islets and exocrine glands became very clear, and the cytoplasm of islet cells increased (Figure 7C). In the group of MFAE treatment, the shape of islets was more complete, the boundary between islets and exocrine glands became clear, the cytoplasm of islet cells increased and the phenomenon of vacuolar degeneration decreased, especially in the high-dose MFAE group (Figure 7D,E), compared with diabetic group.

## Discussion

The rat model of T2DM induced by HSFD and low-dose of STZ simulated the development of human diabetes mellitus (including insulin resistance and islet β cell dysfunction). Compared with spontaneous diabetic mice, this model has abundant animal resources and low experimental cost, so it is widely used in the evaluation of the efficacy of drugs for T2DM [25]. Therefore, HSFD and STZ injection play an indispensable role in the formation of diabetic rat model, and suitable for evaluating the efficacy of antidiabetic drugs.

In the body, there are five ways to remove blood sugar: oxidative decomposition of glucose in various tissues, synthesis of hepatoglycans and myoglycans, conversion into other sugars and derivatives, conversion into non-sugar substances, and excretion from urine when blood sugar concentration is too high. The regulatory effects of various components in MFAE on glucose metabolism have been reported [9,13], and alkaloids represented by 1-DNJ have been the most widely reported. Alkaloids in MFAE can increase the consumption of glucose in hepatocytes, increase the utilization rate of glucose in tissues, and decrease the concentration of blood sugar [13]. In addition, the flavonoids and polysaccharides in MFAE can also regulate blood glucose and prevent complications. In the study, the results confirmed that the hepatic glycogen synthesis of rats in MFAE group was significantly increased, which implied that MFAE can promote the synthesis of hepatic glycogen, improve the utilization of glucose in the liver, play a role in regulating glucose metabolism and reducing fasting blood sugar concentration.

Insulin resistance, as an important pathogenesis of T2DM, is closely related to lipid metabolism disorder, islet β-cell damage and islet failure [26]. Improving insulin resistance is the key to treat and delay the occurrence and development of T2DM. In our study, the result showed that MFAE can reduce insulin concentration, increase insulin sensitivity, and improve insulin resistance symptoms in rats.

In addition, oxidative stress is an important factor in the occurrence of T2DM and its complications. The hyperglycemic metabolic state (including glucose oxidation, polyol metabolism, prostatic synthesis, protein glycosylation) leads to the production of a large number of superoxide and the imbalance of antioxidant function, which eventually leads to tissue damage and a series of diseases [27]. Therefore, the antioxidant effect of reactive oxygen species (ROS) plays an important role in the early stage, progression and complications of diabetes mellitus. In the study, the results showed that MFAE could increase SOD activity, decrease serum MDA content, and prevent oxidative stress from damaging the body. Thus, the ROS pathway could be one of the various mechanisms of MFAE that is beneficial.

Furthermore, the histopathology analysis of pancreas showed that after MFAE treatment, the shape of islets became more complete, the boundary between islets and exocrine glands became clear, the cytoplasm of islet cells increased and the phenomenon of vacuolar degeneration decreased, especially in the high-dose MFAE group, compared with diabetic group. This result displayed a potential therapeutic effect of MFAE, which is worthy of deeply exploring and clarifying the action and mechanism of MFAE.

In conclusion, MFAE exhibited an effective effect on the treatment of T2DM through improving insulin resistance, promoting glycogen synthesis, and improving the body’s antioxidant capacity, thus playing an important role in the potential therapy of T2DM.

## Data Availability

The datasets used and/or analyzed during the current study are available from the corresponding author on reasonable request.

## Abbreviations

HPLC: high-performance liquid chromatography analysis
HSFD: high sugar and high-fat diet
IRI: insulin resistance index
ISI: insulin sensitivity index
MDA: malondialdehyde
MFAE: *Mori folium* aqueous extract
ROS: reactive oxygen species
SD: Sprague–Dawley
SOD: superoxide dismutase
STZ: streptozotocin
T2DM: type 2 diabetes mellitus
1-DNJ: 1-deoxynojirimycin
